# Focus on Function – a randomized controlled trial comparing two rehabilitation interventions for young children with cerebral palsy

**DOI:** 10.1186/1471-2431-7-31

**Published:** 2007-09-27

**Authors:** Mary Law, Johanna Darrah, Nancy Pollock, Peter Rosenbaum, Dianne Russell, Stephen D Walter, Theresa Petrenchik, Brenda Wilson, Virginia Wright

**Affiliations:** 1 *CanChild *Centre for Childhood Disability Research, McMaster University, IAHS Bldg 1400 Main St W., Hamilton, Ontario, L8S 1C7, Canada; 2 School of Rehabilitation Science, McMaster University, IAHS Bldg,1400 Main St W., Hamilton, Ontario, L8S 1C7, Canada; 3 Department. of Physical Therapy, Faculty of Rehabilitation Medicine, University of Alberta Edmonton, AB, T6G 2G4, Canada; 4 Department of Pediatrics, McMaster University, IAHS Bldg, 1400 Main St W., Hamilton, Ontario, L8S 1C7, Canada; 5 Department of Clinical epidemiology and Biostatistics, McMaster University, 1200 Main Street West, Hamilton, Ontario, L8N 3Z5, Canada; 6 Decision Support Research Team, Calgary Health Region, 10101 Southport Road SW Calgary, Alberta, T2W 3N2, Canada; 7 Bloorview Research Institute, 150 Kilgour Road, Toronto, Ontario, M4G 1R8, Canada

## Abstract

**Background:**

Children with cerebral palsy receive a variety of long-term physical and occupational therapy interventions to facilitate development and to enhance functional independence in movement, self-care, play, school activities and leisure. Considerable human and financial resources are directed at the "intervention" of the problems of cerebral palsy, although the available evidence supporting current interventions is inconclusive. A considerable degree of uncertainty remains about the appropriate therapeutic approaches to manage the habilitation of children with cerebral palsy. The primary objective of this project is to conduct a multi-site randomized clinical trial to evaluate the efficacy of a task/context-focused approach compared to a child-focused remediation approach in improving performance of functional tasks and mobility, increasing participation in everyday activities, and improving quality of life in children 12 months to 5 years of age who have cerebral palsy.

**Method/Design:**

A multi-centred randomized controlled trial research design will be used. Children will be recruited from a representative sample of children attending publicly-funded regional children's rehabilitation centers serving children with disabilities in Ontario and Alberta in Canada. Target sample size is 220 children with cerebral palsy aged 12 months to 5 years at recruitment date. Therapists are randomly assigned to deliver either a context-focused approach or a child-focused approach. Children follow their therapist into their treatment arm. Outcomes will be evaluated at baseline, after 6 months of treatment and at a 3-month follow-up period. Outcomes represent the components of the International Classification of Functioning, Disability and Health, including body function and structure (range of motion), activities (performance of functional tasks, motor function), participation (involvement in formal and informal activities), and environment (parent perceptions of care, parental empowerment).

**Discussion:**

This paper presents the background information, design and protocol for a randomized controlled trial comparing a task/context-focused approach to a child-focused remediation approach in improving functional outcomes for young children with cerebral palsy.

**Trial registration:**

[clinical trial registration #: NCT00469872]

## Background

Cerebral palsy comprises a complex, multi-dimensional group of non-progressive movement disorders resulting from damage to the brain prenatally, perinatally, or early in childhood. Incidence of cerebral palsy has remained constant over the past two decades at 2.0 to 2.5 per 1000 live births [1,2]. The long-term disability and costs to the health care system and society associated with cerebral palsy are significant. Cost of services to these children and their families is substantial, with health costs alone estimated at $1,406 per family per year (over $6 billion per year) [3]. Non-reimbursed costs to families for services, equipment, and lost family income can amount to thousands of dollars each year [4]. Honeycutt et al. [5] state that the extra economic lifetime costs associated with cerebral palsy is $800,000 per person.

From the viewpoint of the International Classification of Functioning, Disability and Health (ICF) [6], cerebral palsy presents with "impairments" in body function and structure such as muscle tone, strength, reflexes and range of motion. Significant "activity" limitations can also be present (e.g., dressing, feeding, functional mobility) as well as restricted "participation" (e.g., playing, participating in school) in social and community roles for the child.

This project, coordinated from *CanChild *Centre for Childhood Disability Research at McMaster University, Hamilton, Canada builds on research conducted by our research team over the past several years to develop and test a task/context-focused approach to treatment for young children with cerebral palsy.

The primary objective of this project is to conduct a multi-site randomized clinical trial to evaluate the efficacy of a task/context-focused approach compared to a child-focused remediation approach in improving performance of functional tasks and mobility, increasing participation in everyday activities, and improving quality of life in children 12 months to 5 years of age who have cerebral palsy.

In North America, neurodevelopmental treatment is the most commonly used treatment technique used by therapists in their care of children with a diagnosis of cerebral palsy [7]. Although this approach acknowledges functional independence as an important goal of treatment [8,9], the means to obtain function focuses on remediation of the child in the International Classification of Functioning, Disability and Health (ICF) component of body function and structure. The therapist attempts to inhibit abnormal posture and movement and to improve the child's quality and efficiency of movement by encouraging typical patterns of movement [10,11]. It is assumed that "typical" patterns of movement will lead to functional improvements and reduce activity limitations and participation restrictions. Research to support this assumption is inconclusive [12].

Some therapists and researchers are questioning this emphasis on "normality" because it may not explore all options for functional success [13-17]. Compensatory movements and environmental adaptations may be more efficient solutions to the motor challenges encountered by children with cerebral palsy [18-20]. From this latter perspective, performing the functional task, rather than attainment of normal patterns of movement, is the important goal of treatment.

An array of factors has influenced this philosophical shift – including current models of health status, the perspectives of persons with disabilities, family-centered service delivery, and the application of dynamic systems theory (DST) to motor behavior. Each concept is discussed briefly here.

### (a) Models of health status

Therapists are now using models of health status, such as the ICF [6], as frameworks to identify primary goals, and to evaluate treatment effects. These models have provided a framework by which to discuss and question the assumption of a cause and effect relationship between impairments and functional restrictions [6,21,22]. Functional performance is the result of the dynamic interaction of a myriad of factors, not just those at the level of impairment within the child. The new component of contextual factors in the ICF model encourages therapists to consider the influence of personal and environmental attributes on function simultaneously with the physical abilities of the person. The concept that many factors both internal and external to the child influence functional motor success has caused a re-evaluation of current treatment approaches based on a hierarchical neuromaturational model focused primarily on changing the child's abilities.

### (b) Influence of persons with disabilities

Changing societal attitudes towards disability complement the health status frameworks used in this study. Traditionally, disability was viewed as a problem within the person and the goal was to fix, heal or prevent the problem [23]. Persons with disabilities advocated for a change in this perspective, suggesting that disability is a political rather than a medical issue. The social construction model of disability put forward that society's values and beliefs artificially partition persons into 'disabled' and 'able-bodied' roles in society, and prevent the full participation of persons with disabilities in the community [23-27].

### (c) Family-centered principles

The development of the family-centered philosophy in rehabilitation practice has also influenced attitude changes in the management of children with motor dysfunction. Family-centered service principles clearly articulate that parents know their children best. Family-centered service acknowledges that families are different and unique, and that optimal child functioning occurs within a supportive family and community context [28]. Within this framework, the therapist is viewed as a collaborator, not an expert. Goals of treatment are identified collaboratively with input from the family, child and therapist. This change in service delivery has created an environment conducive to the identification of functional goals at the level of activity and participation rather than exclusively at the level of impairment. There is a need to explore all treatment strategies, including changes to the task and environment, in order to facilitate the achievement of these goals.

### (d) Dynamic Systems Theory

Dynamic Systems Theory (DST) is a recent framework to explain motor development [29,30]. DST suggests that the most efficient motor behavior results from the spontaneous self-organization and interaction of many subsystems to achieve a functional goal. These subsystems derive from three sources: the child, the task and the environment [31]. Within the child, subsystems include not only the central nervous system, but also factors such as biomechanics, anthropometric measures (e.g., head size), temperament, and cognition. Examples of subsystems within the task (what the child is trying to do) which affect motor behavior, are the shape of an object being grasped, or the height of a table that the child uses to pull to stand. In the environment, diverse factors such as the surface on which the child is moving, the effect of gravity and the child's interaction with caregivers or therapists may contribute to the motor behavior that emerges.

The concept that spontaneous self-organization results in the best movement solution challenges therapists to reconsider the traditional therapeutic rejection of "abnormal" movement patterns such as "bunny-hopping" and "W-sitting" that many children with cerebral palsy spontaneously discover and use effectively. Historically, therapists have discouraged these "abnormal" movement patterns because of concern that they would prevent the emergence of more typical ways to move and sit and might result in decreased range of motion. Should these movement solutions be discouraged or accepted as innovative solutions? DST theory challenges traditional treatment perspective that "typical" patterns of movements are the optimal solution for all children. New treatment models are emerging that consider functional success the goal of treatment with less concern about the "normality" of the movement strategy [18,20,32,33].

From a DST perspective, adaptation of the environment and/or task is acceptable as a solution to a motor problem rather than immediately focusing on changing the abilities of the child. Burton and Davis' [34] ecological task assessment is based on matching the task and environment with the abilities of the child in order for the child to achieve success, instead of trying to change the child to conform to an existing environment. For example, rather than viewing walking as the optimal mobility method, some therapists now advocate an array of movement options for children with cerebral palsy that represent the best fit with specific environments [35].

### Integrating these themes – the Task/Context-Focused Approach

The following principles define the key components of the task/context-focused approach.

#### 1. Promote Functional Performance

The goal of treatment is for the child successfully to achieve a specific functional goal that has been identified collaboratively by the family, child and therapist. Emphasis is placed on success with the task rather than the attainment of "normal" patterns of movement. The underlying principle is that there is no one right way to perform a task. Different solutions may be used in different environments. For example, if the goal is for the child to move independently in the home, the child may creep on his or her abdomen on the smooth surface of the kitchen floor but change to rolling on the carpet in the living room. This principle is derived from the DST tenet that movement is always goal-oriented and context specific.

#### 2. Identify Periods of Change (Transition)

Treatment will be most successful if it is introduced at a time when the child is trying to do a new task or attempting to do an established task in a different way. This premise fits with the concept of a developmental, global readiness for performing a new motor task or changing the way an established task is accomplished. Parents (and child) will play an integral role in deciding when the child is ready to attempt a new skill. Transition is a concept of DST that is defined kinematically as the period when a child's movement patterns are more easily perturbed and take longer to return to a stable state. Clinically, the concept of transition is intriguing because it implies a "window of opportunity" when a child is most ready to achieve new functional goals. A study by Trahan & Malouin [36] provides evidence that short term intensive therapy at this "right time" may be as effective as longer term, regular therapy. In a multiple baseline design, five children with cerebral palsy who received short periods of intensive therapy (4 times/week for 4 weeks) followed by no therapy for 8 weeks maintained gains in their motor skills. Using transition as an indicator of readiness, treatment will be time-limited and aimed specifically at success on an identified functional goal.

#### 3. Identify and Change the Primary Constraints

Constraints or rate-limiting factors that prevent achievement of a functional goal are identified. Treatment strategies will be based on the identification of both constraints and enablers of a specific goal by the parents, child and therapist. These constraints and enablers may be identified in the child, the task (goal) or the environment. For each constraint, the therapist must consider if the specific constraint can be changed or whether an adaptation is needed. Examples within the environment are the effects of gravity, space to move, floor surface etc. Examples of how the task may be changed are changing the size of the spoon for a feeding goal and modifying a walker for a mobility goal. Examples of constraints within the child are muscle strength, range of motion, motivation. Constraints/enablers associated with the task and/or the environment will be the first focus of treatment before attempts to change constraints within the child. If a specific changeable constraint is identified within the child, intervention may include changing this constraint through time-limited intervention but only at the level of activity and/or participation. This approach is different from a child-focused approach that centers on remediation of the abilities of the child, usually at the level of body structure and function. The aim of the task/context-focused treatment is to achieve success at the functional goal as soon as possible rather than emphasize the quality of a child's movement.

#### 4. Provide Opportunities for Practice

Children will have opportunities to practice a new skill in the most appropriate environment. Sometimes skills will emerge spontaneously when an appropriate constraint has been changed, but typically in childhood, developmental skills require practice for refinement and to become more automatic. If necessary, practice will be incorporated into the treatment. Again, the emphasis will be on achievement of the functional goal in a natural setting. Practice focuses on the functional goal in the most appropriate environment [37].

### The Child-Focused Approach

A review of the literature in children's rehabilitation in preparation for this project yielded 77 papers reporting research that involved children with cerebral palsy. A wide variety of treatment approaches are being used with this population including surgical treatments such as tendon transfers [38] and dorsal rhizotomy [39,40] and pharmacological treatments, most typically, botulinum toxin injections and intrathecal baclofen [7,41,42].

Treatment approaches utilized by physical and occupational therapists focus primarily on remediation of body function and structure in children with cerebral palsy. These treatments include: maintaining range of motion and joint alignment through stretching, casting and splinting [43,44], strength training [45-47], facilitation of normal movement patterns and postural control through physical handling and practice of functional activities [12,48,49], treadmill training [50] and electrical nerve stimulation [51,52]. Adaptive equipment is frequently prescribed for seating, positioning, mobility and function [53,54]. Only four articles were found that evaluated a functional therapy approach [33,37,55,56].

The therapy literature describing treatment approaches continues to emphasize neurodevelopmental approaches, primarily aimed at changing body function and structure in the child. Treatment now embraces tenets of motor learning and systems theories applied to movement. For example, identification of functional goals and the importance of the child taking control of movements [8] are now both incorporated as important components. However, the means to attain these goals still emphasize changing the child's quality of movement, with typical movement patterns used as the "gold standard" [10,11]. Improvements in functional performance are frequently cited as the desired outcome; however, the methods for achieving this outcome are primarily through changes in body function and structure within the child [57,58].

## Methods/Design

The goal of this randomized controlled cluster design trial is to evaluate the efficacy of the task/context-focused approach with young children ages 12 months to 5 years 11 months with cerebral palsy compared to a child-focused approach to improve performance of functional tasks, mobility, participation in everyday activities, and quality of life. We will recruit a population-based sample of 220 children with cerebral palsy ages 12 months to 5 years 11 months at recruitment date.

The research ethics boards at McMaster University, the University of Alberta, the Calgary Health Region and all participating centres granted approval for the study. The study has a data and safety monitoring plan, which includes a Data and Safety Monitoring Board (DSMB) as per NIH procedures for multi-site clinical trials. All participants will be informed that their participation is voluntary and that they can withdraw at any time without affecting their regular health care. Parents will be asked to read and sign a consent form for the project. The trial intervention period is 6 months in duration and poses no known risks to study participants. One unlikely risk may be that children lose range of motion in certain joints that may ultimately affect their motor function. To ensure that there are no adverse restrictions in range of motion from either treatment, range of motion will be evaluated for all children at baseline, and 3, 6, and 9 months. If a child's range decreases by 8–10 degrees, the absolute magnitude of the current ROM will be examined. If the current absolute value of the range is clinically worrisome, the child's treating therapist will be contacted. He/she will measure the joint again. If ROM remains worrisome, the site coordinator will be notified and will arrange for a physician referral. Any stretching intervention implemented will be recorded and the child will remain involved in the study and included in the analysis, even if therapy is modified.

### Study sample

Children will be recruited from a representative sample of children attending publicly-funded regional children's rehabilitation centers serving children with disabilities in Ontario and Alberta, Canada. Because the Canadian health system enables all citizens to have universal and equal access to publicly-funded services, and because virtually all children with cerebral palsy are served in the programs that will be participating in the study, the sample recruited from the services and programs described above is expected to be fully representative of children with cerebral palsy. 24 centers, 16 in Alberta and 8 in Ontario, will participate.

### Inclusion criteria

▸ Children of either sex with diagnosis of cerebral palsy based on published diagnostic criteria [59]

▸ Children classified in Levels I-V on the Gross Motor Function Classification System (GMFCS) (as determined by their current therapist)

▸ Children aged 12 months to 5 years 11 months at the time of recruitment

▸ Some of the younger children might not yet have an official diagnosis of cerebral palsy. Therapists will be asked to identify suitable children for the study using a screening criteria checklist for children with significant developmental motor delays, which was used in a previous study. These children will have a confirmed diagnosis of cerebral palsy upon exit from the study.

### Exclusion criteria

▸ Children with planned surgical intervention or medication changes during the study period that may affect motor function.

▸ Children whose families feel uncomfortable or unable to respond to interviews and questionnaires in English (the language of virtually all the study materials). Our experience in previous research has shown that less than 5% will be excluded on this criterion.

▸ Since this an efficacy study, parents or caregivers who state that they will not be able to adhere to the treatment schedule will not be entered in the study.

### Sample size

Sample size was calculated for the primary study outcome, using the following: where n = number of subjects per group, **α **= significance level (2-sided testing), **1 - β **= power, **σ **= SD of change within individuals, ICC = intraclass correlation, m = average cluster size, **Δ **= minimum absolute change to be detected, number of clusters (therapists) = n/m. The within-child standard deviation for change on the PEDI scale was set at 7. This value is based on data reported in the PEDI manual, and data from Ketelaar et al. [33]. Specifically, the value 7 is the working average of the within-child standard deviation observed by Ketelaar et al. [33] for self-care functional skills and mobility. We took the average cluster (number of children per therapist) size to be 3, the ICC to be 0.1, and therefore assumed a variance inflation factor (design effect) associated with therapists to be 1.2. The variance inflation factor allows for the possibility that some therapists may be more effective in inducing a change on the PEDI scale compared to other therapists, and takes into account the clustered nature of the sample of participating children. Based on data from our task/context-focused pilot study, we projected an estimated difference in the change scores between the treatment groups of 3 points on the PEDI, and used a two -sided α-value of 0.05, with power of 80%. Our sample size estimates are based on the PEDI because it demonstrates the greatest score variability, and because it is the primary measure of interest in the study. The specifications above lead to a required sample size of 104 children per group, which we rounded to 105 per group so that 35 therapists would be required per group. With additional children to account for dropouts, our target sample size is set at 220.

### Recruitment procedure

Each center in the study has a site coordinator who contacts all therapists to inform them about the study and to obtain their consent to participate. Once therapists have consented to participate in the study they will be block randomized by the coordinating site (by OT or PT discipline) into either treatment arm. On behalf of the research team, each center in the study will mail information that will describe the study to eligible families. Families will be asked to read over the information and indicate their interest in the study on a form to be returned to the research center in a stamped self-addressed envelope. At the baseline visit, study assessors will review the information sheet with the families and witness and co-sign the consent form. Assessors will leave a signed (carbon) copy of the consent form with the family.

Children from consenting families will follow their therapist into the treatment arm that the therapist was randomly assigned to. Due to the nature of the therapy, it will not be possible to keep therapists or parents unaware of the child's treatment group. Therefore, trained, reliable evaluators who are blinded to group assignment will measure all outcomes. Evaluations will be completed at baseline, after 6 months, and at a 3-month follow-up.

### Study treatments

Children in the study will receive either a child-focused therapy approach or the task/context-focused approach for 6 months. All children will return to their regular therapy schedule and approach between 6 months and the 9 month follow-up assessment. Table 1 outlines the primary components of each therapy treatment. Children will receive 18 – 24 sessions of therapy over the 6-month study period. Parent education and consultation about general disability information regarding their child will be provided to both treatment groups.

**Table 1:** Comparison of Process and Components of Child-Focused and Context-Focused Approaches

**Factor**	***Context-Focused Approach***	***Child-Focused Approach***
Location	Home or community setting	Outpatient or community setting

Therapist	Physical or occupational therapist (prime therapist model) Only one therapist providing intervention (other therapists available for consultation and development of treatment plan)	Regular physical therapist and/or occupational therapist May have more than one therapist

Goal Identification	Parent defines goals based on identification of transitions using the COPM	Therapist sets goals after discussion with parent, teachers, etc.

Assessment Process	Observe and videotape child performing identified tasks and use the task/context-focused framework for performance analysis to identify barriers and supports to performance. *"Top-down" approach starting with identification of functional goals and then identifying specific constraints that are barriers to achievement of functional goal*	Assess child's current level of functioning in motor, behavioral, functional areas of performance. *"Bottom-up" approach starting with identification of impairments and then identifying functional limitations*

Primary Treatment Strategy	*First target of intervention is to change constraints identified within the task and/or environment* *Work always at activity and/or participation level in the ICF model*. Use success at task as guide to intervention Different movement strategies are expected in different environments	*First target of intervention is a focus on remediation using neurodevelopmental and other regular therapy techniques at the level of body function and structure* *Work from body function and structure in ICF model thus leading to changes in functional activity* Use typical patterns of movement and hierarchical skills as guide to intervention. The movement strategy chosen is expected to generalize across different environments

Frequency	Intermittent and intense, focused on goals identified (typically 3–4 sessions followed by a break before therapy focuses on another task). 18–24 sessions over 6 months.	Regularly scheduled appointments (typically 3–4 times/month). 18 – 24 sessions over 6 months.

### Child-Focused Approach

In the child-focused approach group, the focus of intervention will be on remediation of the child's abilities through changing the components of body function and structure. Therapists trained for this intervention will receive information regarding current intervention topics. For example, for gross motor intervention, therapists will learn how to establish and modify progressive resisted strengthening programs [60], and training programs to improve gross motor performance [61], gait [62], and function [63] using muscle strengthening techniques. Current principles of neurodevelopmental therapy will also be reviewed [9]. Using neurodevelopmental principles, the child is encouraged to develop more normal movement patterns that will assist him/her in achieving the desired goal. Precise therapeutic handling techniques will be used to improve the child's abilities at the component of body function. Goals will be set with the assumption that changes at the level of body function and structure will affect the child's abilities in activity and participation. Environmental adaptations are usually considered only when remediation of the child's abilities is not successful. Therapists in this arm of treatment will receive up to date information about child-focused intervention approaches appearing in the literature, encouraging them to assume a strong evidence based approach to practice.

### Task/Context-Focused Approach

The task/context-focused approach will follow the principles emerging from dynamic systems theory as described above in the literature review. Motor-based tasks, which a child is beginning to do, trying to do differently or showing an interest in doing but having difficulty accomplishing, will be identified by parents using the Canadian Occupational Performance Measure (COPM) assessment [64]. A videotape of each child performing the identified task will be used as the basis for analysis by the therapist and parents. For each task, the next step in the task/context-focused approach is to identify factors in the task, environment and/or child that are hindering the child's performance of the task. Working with the parents, the therapist will identify these constraints through an analysis of observed task performance. Examples of barriers and facilitators will be discussed in the training sessions but an exhaustive list is not provided. Treatment will focus on changing the identified constraints. Therapists will be instructed to change environment and task constraints first to achieve the functional goal. If a specific changeable constraint is identified within the child, intervention may include changing this constraint through time-limited intervention, but only at the level of activity and/or participation.

Intervention will emphasize practice of tasks in natural environments (e.g. home, preschool). It will not focus on the achievement of motor skills within a hierarchical framework or on the achievement of improved quality of movement. Treatment will assist the child in accomplishing the identified functional task with a minimal amount of treatment. Children will be allowed and indeed encouraged to use any compensatory strategies to achieve functional tasks. Therefore spontaneously chosen movement patterns such as W-sitting, scooting, and pulling to standing using arms will be encouraged if they are effective, least intrusive and acceptable to the child. For the task/context-focused approach, we will employ a prime therapist model – one therapist, either an occupational therapist or a physical therapist, will be assigned to each child and will conduct the intervention for that child (with the other therapist providing consultation).

### Delivery of treatment protocols and therapist training

Therapists in both groups will receive one and a half days of study training – the first half-day will focus on the study protocol and will be done together, with therapists in both arms; the second day will be completed separately for each group and will center on implementing the specific components of the task/context-focused approach or child-focused therapy. Expert consultation will be available for therapists in each treatment group. Therapists will have opportunities to discuss case scenarios and view videos of children. For each experience (scenarios, videos), therapists will practice assessing and intervening using the child-focused approach or the task/context-focused approach. The study has clearly defined the key components of each treatment group. We will monitor intervention protocols on an ongoing basis throughout the trial to ensure procedural reliability; as well, the therapists will have access to a consultant, study investigators, and study coordinator for support, problem solving, and resources. Therapists in both treatment groups will complete a short log after each session to document the therapy given. During the study, one therapy session per therapist in each arm will be randomly selected for recording by videotape. These tapes will be evaluated for procedural reliability by trained raters who are occupational therapists or physical therapists. A web-based forum will be developed to encourage regular communication between therapists, the research team and study consultants.

Attendance at therapy appointments will be monitored; those missing a session will be phoned immediately so that another appointment can be scheduled. To measure co-intervention, questionnaires completed by parents and therapists will be used to detail the child's participation in other activities or therapies. As well, we will record any significant illnesses of the children during the 6 month intervention period. If the number of co-interventions or illnesses is not comparable across groups, these data will be included in the analysis as a covariate.

### Measure and data collection procedures

The primary outcome in this study is performance of functional tasks (mobility and self-care) as measured by the Pediatric Evaluation of Disability Inventory (PEDI) [65]. Secondary outcomes include range of motion, gross motor function, participation in everyday activities, health status, parent empowerment, and parents' perceptions of care. Independent evaluators who are unaware of the child's treatment assignment will assess the children and parents/caregivers in their home. Outcome measures in the trial include:

### Body functions and structures (Impairment)

#### 1. Range of motion measure

The independent, trained assessors will take goniometry measurements of hip abduction, popliteal angle, and ankle dorsiflexion ranges of motion of children in both treatment groups. To reduce measurement error, two consecutive measurements will be done of each range of motion and the average calculated.

#### 2. Gross Motor Function Classification System (GMFCS)

The Gross Motor Function Classification System (GMFCS) [66] will be used to classify the gross motor function of children and stratify children based on severity (less severe, levels I and II; more severe, levels III-V). The GMFCS has evidence of very good reliability and validity.

### Activities

#### 1. Pediatric Evaluation of Disability Inventory (PEDI)

The Pediatric Evaluation of Disability Inventory (PEDI) [65] will be used to measure mobility and self-care. The PEDI can be completed by structured interview. Part I: Functional Skills and Part II: Caregiver Assistance will be administered for the Mobility and Self-care Domains. Internal consistency measured by Cronbach's alpha is .99 for the Self-Care domain and .97 for the Mobility domain. Inter-rater reliability (ICC) between interviewers is 1.00 for both domains among rehabilitation therapists; and .88 and .98 when completed by different family members. Evidence of concurrent validity is provided by the high correlations between the Self-Care domain and Wee-FIM Self-Care scores (r = .92) and Mobility domain and Wee-FIM Mobility scores (r = .97). Responsiveness of the PEDI for children with CP was demonstrated over a 4–6 month period for the Self-care and Mobility Domains when assessed by a therapist and in the Self-care Domain when assessed by a parent [67].

#### 2. Gross Motor Function Measure (GMFM-88)

The 88-item version of the GMFM includes five dimensions (Prone and Supine; Sitting; Crawling and Kneeling; Standing; Walking, Running and Jumping). Each item is measured by observation and scored on a 4-point ordinal scale. The GMFM is the gold-standard measure used in clinical trials for children with cerebral palsy. Validation of the GMFM for children with cerebral palsy was first published in 1989 [68]. Additional evidence of validity and responsiveness of the GMFM for children with spastic diplegia and quadriplegia has also been established [69]. Several studies have documented the test-retest and inter-rater reliability of the 88-item GMFM [69-71]. Inter-rater reliability ranges from 0.87 to 0.99 and test-retest reliability ranges between 0.76 to 0.99

### Participation

#### 1. Pediatric Quality of Life Inventory ~ PedsQL 3.0 Cerebral Palsy Module

The PedsQL 3.0 CP Module was designed to measure HRQoL dimensions specific to CP [72]. The CP Module is divided into two components: parent report for toddlers aged 2–4 years; and a child self-report and parent report of children aged 5–7, 8–12 and 13–18 years old. The parent report for toddlers contains 22 items. These 22 items are split into 5 dimensions comprised of daily activities, movement and balance, pain and hurt, fatigue and eating activities. Psychometric properties for the PedsQL 3.0 CP Module were derived from a study with a sample of children with Cerebral Palsy ages 5–18 years [72]. Construct validity was supported for the CP Module and a reliability of was reached at 0.79 for child report and 0.91 for parent report using Cronbach's alpha [72].

#### 2. Children's Assessment of Participation and Enjoyment (CAPE)

The CAPE is a measure developed at *CanChild *for a study of children's participation and is designed to assess children's participation in voluntary, day-to-day activities outside of mandated school activities [73]. The CAPE uses pictures to help children identify what activities they do. For each identified activity they are asked several questions: how often they have done the activity in the past 4 months, with whom they typically do the activity; where they do the activity and how much they enjoy the activity. This measure has been used successfully with over 400 children in the "Participate" study. Test-retest reliability of the CAPE scales ranges from 0.80 – 0.90. Scores on the CAPE correlate significantly with child's communication, social and physical function.

### Environment

#### 1. Family Empowerment Scale (FES)

Koren, DeChillo and Friesen [74] designed the Family Empowerment Scale (FES) to measure this construct. Singh et al. [75] performed a factor analysis on the FES revealing four dimensions: systems advocacy, knowledge, competence and self-efficacy. The FES has been used in a number of efficacy studies in the fields of children's mental health [76,77], diabetes (Florian & Elad, 1998), and low-income families [78]. The FES has excellent reliability (Internal consistency 0.87 – 0.88; test-retest reliability 0.77–0.85) and evidence of discriminative validity.

#### 2. Measure of Processes of Caregiving (MPOC-20)

MPOC was developed to measure the processes of professional caregiving [79]. MPOC is a 20-item questionnaire with five constructs 1) Enabling & Partnerships; 2) Providing General Information; 3) Providing Specific Information about Child; 4) Coordinated and Comprehensive Care for the child and family; 5) Respectful and Supportive Care. MPOC has been used in a study of factors affecting the health of caregivers of children with disabilities [80]. Internal consistency was examined using 3 data sets with Cronbach's alpha ranging from 0.81–0.96. Test-retest reliability (n = 29) had ICC values ranging from 0.78–0.88 [81]. Reliability and validity of the MPOC are excellent.

### Analyses

All data will be stored in a secure environment and analysed at the McMaster coordinating site. Data from each outcome assessment will be collected and summarized for each treatment group. Descriptive statistics including frequencies, means, and standard deviations will be calculated. To test for the effect of the intervention, the differences between the means for the task/context-focused group and the regular therapy care group will be evaluated. The primary analysis will be to estimate the differences in the change scores between groups, comparing baseline to both the 6-month intervention and 3-month follow-up intervals. A combined analysis for the 3 time points will use a mixed effects repeated measures analysis of variance (ANOVA) linear model. Treatment groups and time will be taken as fixed factors, while the clusters (therapists) will be regarded as a random factor nested within treatment groups [82].

We anticipate that the sample sizes will be approximately balanced between the intervention groups, but if unequal sample sizes occur for some reason, generalized least squares techniques will be used for the statistical analysis. The primary focus will be on the group × time interaction, for which the cluster × time interaction will be used as an error term for testing significance. The statistical significance level will be set at 0.05 for the primary outcome (PEDI assessment). Results will be presented in terms of the estimated mean difference in change between the treatment groups, together with 95% confidence intervals. For secondary outcomes, we will again report means and confidence intervals, but we will be more cautious in our interpretation of these results, recognizing that a large number of statistical comparisons will be made, and also that our power for secondary outcomes may not be as great as for PEDI. Prognostic variables such as age, center and co-intervention, if shown to be important predictors of outcome, will be included in the analysis as covariates. Statistically significant interactions would imply that the treatment effects differ between age and/or severity subgroups. If such interactions are significant, individual contrasts will be examined to determine if there are age/severity subgroups in which treatment differences are statistically and clinically important. An intention to treat analysis will be employed – those who do not adhere to treatment (miss >20% of sessions) will be included in the analysis in their original group. Children who drop out of the study will be included in the analysis in their original group if outcome data can be collected.

## Discussion

In this paper, we have presented the background and design for a randomized controlled trial comparing a task/context-focused treatment approach to a child-focused treatment approach for young children with cerebral palsy. In light of several exciting conceptual innovations regarding motor development of young children, and in approaches to the delivery of care and services to families of children with disabilities, we believe the time is right for a study of the size, scope and nature of this trial. Whatever the outcome, the results will be widely generalizable and applicable, and will contribute importantly to our understanding of the management of a complex and prevalent child health problem. The findings from this study will be widely disseminated through traditional methods such as publications in peer reviewed journals and academic conferences, in addition to CanChild's website and communication through parent networks. As well, all families whose children participated in the study will receive an easy-to-read summary of the study findings.

## Abbreviations

NIH: National Institutes of Health

ICF: International Classification of Functioning, Disability and Health

DST: Dynamic Systems Theory

DSMB: Data and Safety Monitoring Board

ROM: range of motion

GMFCS: Gross Motor Function Classification System

ICC: intraclass correlation

PEDI: Pediatric Evaluation of Disability Inventory

OT: Occupational Therapy/Therapist

PT: Physiotherapy/Physiotherapist

COPM: Canadian Occupational Performance Measure

GMFM: Gross Motor Function Measure

PedsQL: Pediatric Quality of Life Inventory

CP: cerebral palsy

HRQoL: health related quality of life

CAPE: Children's Assessment of Participation and Enjoyment

FES: Family Environment Scale

MPOC: Measure of Processes of Care

ANOVA: analysis of variance

## Competing interests

The author(s) declare that they have no competing interests.

## Authors' contributions

ML, JD, PR, SW, NP and DR were responsible for the design of the study proposal. TP, BW and VW are involved in the study implementation. All authors participated in the writing and/or reviewing of this manuscript.

## Pre-publication history

The pre-publication history for this paper can be accessed here:


